# Upregulation of Early and Downregulation of Terminal Pathway Complement Genes in Subcutaneous Adipose Tissue and Adipocytes in Acquired Obesity

**DOI:** 10.3389/fimmu.2017.00545

**Published:** 2017-05-16

**Authors:** Sanna Kaye, A. Inkeri Lokki, Anna Hanttu, Eija Nissilä, Sini Heinonen, Antti Hakkarainen, Jesper Lundbom, Nina Lundbom, Lilli Saarinen, Olli Tynninen, Maheswary Muniandy, Aila Rissanen, Jaakko Kaprio, Seppo Meri, Kirsi H. Pietiläinen

**Affiliations:** ^1^Obesity Research Unit, Research Programs Unit, Diabetes and Obesity, University of Helsinki, Helsinki, Finland; ^2^Helsinki Haartman City Hospital, Department of Emergency Care, Helsinki, Finland; ^3^Department of Bacteriology and Immunology, University of Helsinki and Helsinki Central Hospital, Helsinki, Finland; ^4^Immunobiology Research Program, Research Programs Unit, University of Helsinki, Helsinki, Finland; ^5^Department of Medical and Clinical Genetics, Helsinki University Central Hospital, University of Helsinki, Helsinki, Finland; ^6^HUS Medical Imaging Center, Radiology, Helsinki University Hospital, University of Helsinki, Helsinki, Finland; ^7^Genome-Scale Biology Research Program, Research Programs Unit, University of Helsinki, Helsinki, Finland; ^8^Department of Pathology, University of Helsinki and HUSLAB, Helsinki University Hospital, Helsinki, Finland; ^9^Department of Public Health, University of Helsinki, Helsinki, Finland; ^10^Department of Psychiatry, Helsinki University Central Hospital, Helsinki, Finland; ^11^Institute for Molecular Medicine FIMM, University of Helsinki, Helsinki, Finland; ^12^Obesity Center, Endocrinology, Abdominal Center, Helsinki University Central Hospital and University of Helsinki, Helsinki, Finland

**Keywords:** obesity, complement system, gene expression, twin study, monozygotic twins

## Abstract

Inflammation is an important mediator of obesity-related complications such as the metabolic syndrome but its causes and mechanisms are unknown. As the complement system is a key mediator of inflammation, we studied whether it is activated in acquired obesity in subcutaneous adipose tissue (AT) and isolated adipocytes. We used a special study design of genetically matched controls of lean and heavy groups, rare monozygotic twin pairs discordant for body mass index (BMI) [*n* = 26, within-pair difference (Δ) in body mass index, BMI >3 kg/m^2^] with as much as 18 kg mean Δweight. Additionally, 14 BMI-concordant (BMI <3 kg/m^2^) served as a reference group. The detailed measurements included body composition (DEXA), fat distribution (MRI), glucose, insulin, adipokines, C3a and SC5b-9 levels, and the expression of complement and insulin signaling pathway-related genes in AT and adipocytes. In both AT and isolated adipocytes, the classical and alternative pathway genes were upregulated, and the terminal pathway genes downregulated in the heavier co-twins of the BMI-discordant pairs. The upregulated genes included C1q, C1s, C2, ficolin-1, factor H, receptors for C3a and C5a (C5aR1), and the iC3b receptor (CR3). While the terminal pathway components C5 and C6 were downregulated, its inhibitor clusterin was upregulated. Complement gene upregulation in AT and adipocytes correlated positively with adiposity and hyperinsulinemia and negatively with the expression of insulin signaling-related genes. Plasma C3a, but not SC5b-9, levels were elevated in the heavier co-twins. There were no differences between the co-twins in BMI-concordant pairs. Obesity is associated with increased expression of the early, but not late, complement pathway components and of key receptors. The twins with acquired obesity have therefore an inflated inflammatory activity in the AT. The results suggest that complement is likely involved in orchestrating clearance of apoptotic debris and inflammation in the AT.

## Introduction

The complement system has a pivotal role in obesity. While it is an important innate immune defense system against microbes and part of the body’s clearance system, it can also regulate the level of inflammation in the adipose tissue (AT) and have metabolic effects. Complement is a key innate sensor between viable and non-viable cells. It recognizes and opsonizes both foreign targets, like microbes, and exposed or damaged endogenous structures, and promotes their clearance by macrophages. Altogether, the complement system comprises up to 50 proteins that are synthesized by several tissues including the liver and various cell types of AT (adipocytes, macrophages, and vascular cells) (1–3). The complement proteins circulate in blood as activating or regulating components or act on cell membranes as receptors or protective molecules. Intriguingly, many links exist between the complement system and AT, but overall, the meaning and relevance of these links are mostly unclear.

The complement system can be activated through the classical, the lectin, or the alternative pathway. Specifically, the classical and the lectin pathways can recognize targets with specific pattern recognition molecules C1q, mannan-binding-lectin (MBL), and ficolins 1–3 (FCN1–3) (1–3). In contrast, the alternative pathway is continuously active and can amplify efficiently complement activation against non-self targets (2–4). All three pathways merge to activate the complement component C3, the most abundant complement factor in human blood plasma. Factor D (CFD, adipsin) is synthesized solely by adipocytes and its plasma levels are decreased in obesity (5). Factor D activates complement factor B (CFB) allowing the generation of the alternative pathway C3 convertase C3bBb, which enzymatically cleaves new C3 molecules to C3a and C3b (1–3). Opsonization by C3b and by its inactivated product iC3b leads to the phagocytosis of target structures (1, 3). Activation of the early pathways of the complement system results in the generation of C5a, a potent anaphylatoxin, chemoattractant, and leukocyte activator. Together with C3a, C5a can attract cytokine-producing macrophages and other leukocytes to the area of microbial invasion or tissue damage (1, 3). The completion of the cascade, the terminal pathway, leads to formation of the membrane attack complex (MAC, C5b-9) that allows sodium and calcium influx into cells. Membrane leakage will thus sequentially lead to strong metabolic activation, injury, and ultimately death of the target cell (4). Soluble complement inhibitors such as factor H (CFH), vitronectin and clusterin, and a group of membrane-bound regulators [complement receptor 1 (CR1)/CD35, CRIg, MCP/CD46, DAF/CD55, and protectin/CD59] protect normal viable human cells against complement attack (3).

The complement system includes many proteins relevant to obesity, e.g., acylation-stimulating protein (ASP, C3a desArg) and adipsin (factor D). In addition, the activation-initiating complement proteins are related to adiponectin, which is an insulin-sensitizing and anti-inflammatory adipokine. Its plasma levels decrease in obesity (6). Adiponectin has structural similarity to complement collectins C1q, MBL, surfactant proteins SP-A and SP-D, and FCN1–3. They are all multimers of triple-helical structures, where each subchain has subunits composed of a globular and a collagen-like domain (7). Additionally, adiponectin participates in the clearance of dying cell material by macrophages via a calreticulin-mediated mechanism (8) and is an immune-modulator lowering tumor-necrosis factor-alpha (TNF-α)-levels (9). ASP is identical to the C3 activation product C3a desArg (10). In AT, ASP stimulates glucose uptake and free fatty acid storage postprandially (10). In the complement system, C3a desArg is mostly devoid of the anaphylatoxin or chemotactic activity of C3a. While C3a binds to the C3a receptor (C3aR), C3a desArg binds to the C5a receptor type 2 (C5aR2), which often mediates anti-inflammatory effects (11). C3aR expression has been shown to become upregulated in AT following a high-fat diet (12). It is intriguing that the C3a-C3aR (inflammation) and C3a desArg-C5aR2 (fat and glucose metabolism) interactions have such different effects.

During the progression of obesity, the adipocyte size increases, which associates with inflammation (13) and is linked to the development of insulin resistance (14, 15). Plasma levels of C3, CFH, and CFB correlate positively with body mass index (BMI), waist circumference, triglycerides, and inflammatory parameters and negatively with insulin sensitivity and HDL cholesterol (16, 17). Complement components such as C3 and factor B are overproduced by activated adipocytes in type 2 diabetes mellitus (T2DM) (18), and C3 is among the major determinants of metabolic syndrome in obese patients (19). Thus, the activated complement system is involved in the pathogenesis of cardiovascular disease (20). Upregulated complement gene expression by adipocytes from subcutaneous (sc) fat associates with insulin resistance (21, 22). In a population-based cohort study, plasma levels of C3 were found to correlate positively with plasma insulin and glucose and to associate with the development of type 2 diabetes (23). Since complement is a main mediator of inflammation and clearance of non-viable tissue components, its role in obesity-related fat overload and metabolic disturbances merits more attention.

In humans, most of the studies on the complement system have been conducted in unrelated individuals without control for genetic variation. It is evident that genetic effects have a strong influence on both how complement activation is linked to inflammation (24) and on the weight gain (25) as well as for the developing complications thereafter. To exclude confounding due to shared genetic factors, we undertook a unique approach of analyzing complement gene expression in subcutaneous AT of genetically identical twins either concordant or discordant for obesity. To exclude the effect of the immune-cell-rich stroma-vascular fraction, we further analyzed the complement gene-expression profiles of isolated adipocytes. In addition to comparing the co-twins (effects of acquired obesity), we wanted to know whether complement gene expression is associated with adipocyte size, fat distribution (subcutaneous, intra-abdominal, and liver fat), systemic inflammation, systemic markers of insulin resistance, or the expression of insulin-signaling genes. This was explored separately in the AT and isolated adipocytes from the twin individuals. Finally, we stained AT biopsies immunohistochemically to investigate the localization and intensity of C1q, the key activator of the classical pathway and of C3d, an indicator of prolonged complement activation. The results reveal an unprecedented coupling of the complement recognition molecules and early activation pathways with acquired obesity and related metabolic disturbances.

## Materials and Methods

### Participants

The study population has been described in our previous studies (26–28). Briefly, the monozygotic (MZ) twin pairs (aged 22–36, 54% women) were identified through the national population registry of Finland from 10 population-based birth cohorts (FinnTwin12 and FinnTwin16); 26 pairs being discordant for BMI, within-pair difference (Δ) in BMI >3 kg/m^2^ (mean Δweight 18 kg), and 14 pairs concordant for BMI (ΔBMI <3 kg/m^2^) acting as their controls. All pairs were of European ancestry (Finnish), normotensive, and did not use any other medications than oral contraceptives, except for one obese co-twin with T2DM who used metformin and insulin, and one obese co-twin with inactive ulcerative colitis who used mesalazine and azathioprine. Twenty-three subjects were habitual smokers. AT biopsies were available from all 80 subjects. mRNA was available for gene expression analyses in isolated adipocytes from 38 subjects (14 BMI-discordant and 5 concordant pairs). The clinical characteristics within these twin pairs did not differ from the entire group of 80 twins (Table S1 in Supplementary Material). The protocol was designed according to the principles of the Helsinki Declaration and all subjects gave their written informed consent. The Ethical Committee of the Helsinki University Central Hospital (DNRO 270/13/03/01/2008) approved the protocol.

### Serum and Plasma Analyses, Body Composition, AT Biopsies, and Adipocyte Morphology

Venous blood samples were obtained from all subjects after overnight fasting. EDTA plasma and serum samples were separated by centrifugation and stored at −80°C until the analyses of plasma glucose (spectrophotometric hexokinase and glucose-6-phosphate dehydrogenase assay, Roche Diagnostics, Basel, Switzerland), serum insulin (by time-resolved immunofluorometric assay; Perkin Elmer, Waltham, MA, USA), plasma adiponectin and adipsin (DuoSet ELISA, R&D Systems Europe Ltd., Abingdon, UK), and serum high-sensitive C-reactive protein [hsCRP, Cobas CRP (Latex) HS, Roche Diagnostics] were performed. Plasma levels of fluid phase activation products of the complement system C3a (cleaved from C3) and SC5b-9 (soluble C5b-9) were determined by an enzyme-linked immunosorbent assay using MicroVue C3a Plus and C5b-9 Plus enzyme immunoassay kits (Quidel Corporation, San Diego, CA, USA) according to the manufacturer’s instructions. The absorbances were read at 450 nm in a FluorStar Optima multidetection microplate reader (BMG Labtech GmbH, Ortenberg, Germany), and the concentrations analyzed using Optima’s MARS 2.0 analysis software.

Body composition of the subjects was measured by Dual-energy X-ray absorptiometry (Lunar Prodigy, Madison, WI, USA, software version 8.8). Fat percentage was calculated as fat mass/(fat mass + lean mass + bone mineral content) for the total body. Magnetic resonance imaging (MRI) was used for measuring the volumes of abdominal subcutaneous and intra-abdominal fat, and magnetic resonance spectroscopy for measuring the liver fat percentage, as described earlier (29).

Adipose tissue biopsies were obtained from periumbilical subcutaneous fat using a surgical technique. RNA was prepared from frozen fat tissue (26). Adipocytes were isolated from the fat biopsy specimens treated with collagenase (28). The diameter of fat cells was measured under a light microscope. Fat-cell volume was calculated as described by Heinonen (13) assuming the adipocytes as spheres.

### Transcriptomics Analyses of AT and Adipocytes

Transcriptomics experiments were performed with Affymetrix U133 Plus 2.0 chips. Raw gene expression data were preprocessed with the GeneChip robust multiarray averaging (GCRMA) algorithm using BioConductor (30) in R and by using Brainarray custom CDF files for probe annotations (31). The data were validated with RT-qPCR as described (32). We selected 46 representative genes coding for components of the complement system for the expression analyses in the AT (Table S2 in Supplementary Material). The following genes from the insulin signaling pathway were used in analyses of the relationships between complement system and insulin sensitivity in AT: insulin receptor (INSR), insulin-receptor substrate proteins 1–2 (IRS1–2), and phosphatidylinositol-4,5-bisphosphate 3-kinase, catalytic subunit alpha (PIK3CA). The following complement factors were not included as they were not included in the array (C1QTNF4, C1QTNF6, C4, MBL, MASP-2) or were expressed at low levels (C8, C9, C4BP, C5aR2, and vitronectin) thereby lacking the inter-individual variation. GLUT4 gene was expressed at the lowest detection level, lacked inter-individual variation, and was therefore excluded from AT analyses.

Isolated adipocyte transcriptomics experiments were performed with Affymetrix U133 Plus 2.0 chips similarly as for the AT samples. The expression levels of L-selectin (SELL) and C5aR2 were not found from adipocyte transcripts. The same insulin signaling pathway-related genes than from AT and GLUT4 gene transcripts were analyzed.

### Immunohistochemical Staining

We selected two BMI-discordant pairs and two BMI-concordant control twin pairs to visualize complement proteins in AT. Paraffin blocks of the AT were sectioned at 5 µm and two sections were placed on each slide. One of the sections was used for staining with a primary and secondary antibody, while the other served as a mock control with buffer pipetted in place of the primary antibody.

First, a standard de-paraffination procedure was applied consisting of 2× xylene (5′), followed by a series of ETOH (2′ each) in decreasing concentration and ending up in distilled water where samples slides were stored until the following day in preparation for staining. An antigen retrieval heat-treatment protocol followed, where section slides in EDTA buffer pH8.5 from Sigma (product# E1161, Sigma-Aldrich, St. Louis, MO, USA) + 0.05% Tween were brought to a boiling point in a microwave oven for 15′ followed by cooling in RT for 30′. The actual staining was carried out using 3× PBS washed heat-treated slides in a humidity chamber using Bright Vision plus Poly-HRP-Anti MS/RB/RT IgG REF DPVB55-HRP kit (Immunologic, Duiven, Netherlands). The grease pen limited section was subjected for Hydrogen Peroxide Block (UltraVision; Thermo Fisher Scientific, Waltham, MA, USA) incubation for 10′, where after the manufacturer’s protocol for the staining kit was followed. The primary antibodies were diluted 1:1,000 in PBS (C1q and C3d both Rabbit pAb by DAKO, Glostrup, Denmark). The final step of the staining protocol was counterstaining by Gills Hematoxylin (2″) rinsing (10′) and finally series of ETOH (2′) treatments in increasing concentration finishing in 2× xylene (2′) and mounting by Depex. The slides were dried ON in a fume hood and stored in RT. Tissue histology was confirmed by standard HE staining for each tissue sample. Images of immunohistochemistry were collected using Olympus DP Manager (ver. 2.2.1.195) and Olympus DP Controller (ver. 2.2.1.227) image capture softwares with Olympus BX51 fluorescence microscope camera with 100×, 200×, and 400× magnifications.

### Statistical Analyses

The statistical analyses were performed using Stata statistical software (release 13.0; Stata Corporation). In addition to controlling the effect of gender, the comparison of MZ co-twins (almost 100% identical at the DNA sequence level) allows controlling for genetic and shared environmental effects. Thus, any differences within twin pairs are by definition acquired (in our case, through BMI difference). Comparisons of the heavier vs. leaner co-twins’ clinical characteristics were analyzed by paired *t*-test (normally distributed) or Wilcoxon’s test (non-normally distributed variables). Within-pair comparisons of the log^2^-transformed gene expression values were tested by paired *t*-test.

Student’s *t*-test was used for the co-twin comparison of the gene expression data. In addition, we assessed differences between genders, and smokers and non-smokers (individual twins) with Mann–Whitney *U*-test. Since some differences in the gene expression between smokers vs. non-smokers emerged, within-pair analyses were performed first among all twin pairs, whereafter habitual smokers were removed from the leaner vs. heavier comparison to assess whether the effect of smoking was confounding the results. As the differences in the gene expression between leaner and heavier co-twins still emerged to the same extent before and after exclusion of the smokers, we present the results from the whole cohort, with smokers included. Within-pair analyses are by design adjusted for gender.

Mann–Whitney *U*-test was used to compare whether the characteristics of the leaner and the heavier co-twins in the BMI-discordant pairs were the same in the whole group for whom AT was available and in those for whom data on adipocytes were available.

Using twins as individuals, the partial correlation analyses were performed to determine the relationships between AT/adipocyte gene expression and adiposity measures, adipokines, and the insulin signaling gene expression. Since differences between genders and smokers vs. non-smokers emerged in individual twins’ gene expression, their effect was adjusted in correlation analyses. Logarithmic corrections were performed for log-normally distributed variables prior to the correlation analyses. Subsequently, the *p* values of partial correlates were adjusted for multiple testing by false-discovery rate (FDR) (Benjamini–Hochberg) (33).

## Results

### Study Population

Table 1 summarizes the subjects’ anthropometric and clinical characteristics (26–28). In brief, the heavier co-twins of the discordant pairs weighed on average 18 kg more, had 28% more total fat, 67% more subcutaneous fat, 200% more intra-abdominal fat, and 300% more liver fat than their leaner counterparts. Accordingly, the adipocyte volumes of the heavier co-twins were 57% larger than those of the lean co-twins. Furthermore, the heavier co-twins had higher plasma levels of insulin and adipsin, and lower adiponectin levels. BMI-concordant pair co-twins did not differ in any of the metabolic measures. They were also similar for all complement-related measures, which is why we report only the results from the discordant pairs.

**Table 1:** **The clinical characteristics of the monozygotic twin pairs**.

	BMI-discordant pairs			BMI-concordant pairs (*n* = 14)	

	ΔBMI > 3 kg/m^2^, *n* = 26 pairs			ΔBMI < 3 kg/m^2^, *n* = 14 pairs	
Age (mean years ± SD)			30.4 ± 4.2		
Sex	9 males/17 females			5 males/9 females	
	**Leaner** **Mean ± SE**	**Heavier** **Mean ± SE**	***p***	**Leaner** **Mean ± SE**	**Heavier** **Mean ± SE**
*N* of current smokers	7	7		4	5
BMI	25.28 ± 0.89	31.25 ± 1.02	<0.0001	26.20 ± 0.92	27.65 ± 1.01
Fat percentage (%)	32.25 ± 1.81	41.14 ± 1.32	<0.0001	28.56 ± 2.57	29.89 ± 2.39
Adipocyte volume (μm^3^)^a^	371.9 ± 34.05	584.4 ± 49.55	<0.0001	364.7 ± 54.11	403.1 ± 46.87
Subcutaneous fat (cm^3^)	3,814 ± 416.9	6,359 ± 540.4	<0.0001	3,084 ± 351.7	3,428 ± 394.0
Intra-abdominal fat (cm^3^)	790.2 ± 179.0	1,644 ± 247.4	<0.0001	1,037 ± 173.2	1,093 ± 200.3
Liver fat%	1.12 ± 0.32	4.52 ± 1.00	<0.0001	1.99 ± 0.90	3.75 ± 1.75
fP-insulin (mU/L)^b^	4.93 ± 0.51	8.50 ± 1.21	<0.001	5.34 ± 1.13	5.61 ± 0.60
fP-glucose (mmol/L)^b^	5.13 ± 0.07	5.28 ± 0.11	0.174	5.27 ± 0.11	5.44 ± 0.15
hsCRP (mg/L)	2.56 ± 0.70	4.02 ± 1.14	0.065	1.25 ± 0.55	1.11 ± 0.19
Adipsin (pg/L)^a^	1,192 ± 49.05	1,309 ± 47.34	0.006	1,011 ± 121.7	1,047 ± 81.97
Adiponectin (ng/L)	3,842 ± 284.9	2,820 ± 232.2	0.0001	3,370 ± 539.9	2,603 ± 307.9
fP-C3a (ng/mL)	69.20 ± 4.16	77.82 ± 4.57	0.016	67.70 ± 9.24	61.45 ± 3.28
fP-SC5b-9 (ng/mL)	184.6 ± 10.28	193.64 ± 9.00	0.209	187.0 ± 15.13	184.3 ± 13.19

*Wilcoxon signed-ranks test (leaner vs. heavier twin). Within-pair difference BMI >3 kg/m^2^ in discordant pairs (*n* = 26 pairs, 9 males), BMI <3 kg/m^2^ in concordant pairs (*n* = 14 pairs, 5 males)*.

*BMI, body mass index; fP, fasting plasma; hsCRP, high-sensitive C-reactive protein; C3a, complement component 3a; SC5b-9, the soluble terminal complement complex*.

*^a^n = 8 concordant pairs*.

*^b^25 discordant pairs*.

### The Early Complement Pathway Components Are Upregulated and C3 Is More Activated in Heavier Co-twins

**Figure 1:**
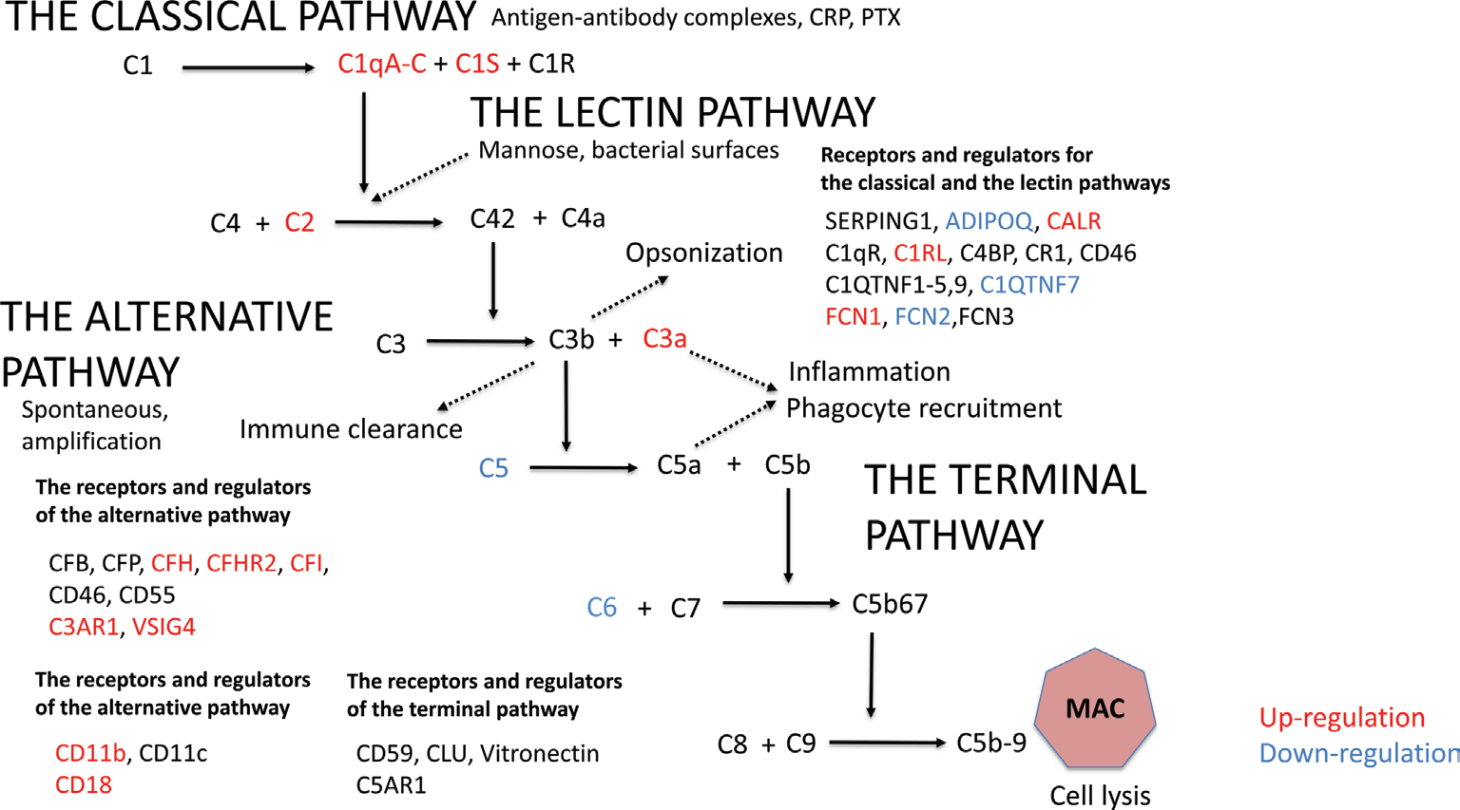
**The gene expression profile in subcutaneous adipose tissue within monozygotic body mass index-discordant twin pairs (heavier vs. leaner) differed in 20 out of 46 genes that code for components of the complement system**. Genes that are upregulated in heavier co-twins are indicated with red and downregulated genes with blue.

Initially, we analyzed the effect of obesity on complement gene expression. In AT, 20 out of the altogether 46 complement genes expressed differently between the leaner and the heavier co-twins of the BMI-discordant pairs (Figure 1; Table S2 in Supplementary Material). In heavier co-twins of BMI-discordant pairs, the C1qA-C genes, those of the classical pathway activation initiating proteins, were upregulated. Accordingly, the genes for other classical pathway components: C1s, the key catalytic component of C1, the receptor for C1q (C1QR), C1r-like protease (C1RL), and C2 were upregulated. In contrast, the genes of FCN2, coding a protein recognizing acetylated carbohydrates in the lectin-pathway, and C1QTNF7, a C1q and TNF-related protein (CTRP) family member, were downregulated in heavier co-twins.

The gene expression of the most abundant complement inhibitor factor H (CFH) of the alternative pathway was upregulated in heavier co-twins compared to their leaner counterparts. Additionally, complement receptor of the immunoglobulin superfamily (CRIg/VSIG4) was upregulated. Thus, while the early classical pathway components were upregulated, the result suggests that complement activation would continue only up to the C3/C5 convertase level because of upregulation of the main inhibitory control factor CFH. In accordance, the plasma levels of C3a and adipsin were higher in the heavier, but the levels of SC5b-9, a marker of terminal pathway activation, were similar between the heavier and the leaner co-twins of BMI-discordant pairs (Table 1). This indicates that complement activation in obese individuals indeed occurred up to the C3 activation level but not beyond that to the terminal pathway.

### Upregulated Gene Expression of Complement Receptors and Complement Inhibitors and Downregulation of the Terminal Pathway in AT of Heavier Co-twins

The genes for C3aR1 and C5aR1, important complement receptors found, e.g., on the surfaces of macrophages and other immune cells, were upregulated in heavier co-twins’ AT (Figure 1; Table S2 in Supplementary Material). Furthermore, the heavier co-twins had an upregulated gene expression of components of the beta-integrin CD11b/CD18, the major phagocytic receptor (CR3) for particles coated with iC3b. The genes of the terminal pathway components C5 and C6 were downregulated, whereas the gene of the soluble terminal complement complex binding clusterin (CLU) was strongly upregulated in heavier co-twins. This indicates that the terminal pathway is suppressed in obese individuals, whereas the receptors for early activation pathway components are upregulated to mediate their functional effects.

### Complement Gene Expression Profiles of Isolated Adipocytes Differ between BMI-Discordant Co-twins

As AT is a mixture of adipocytes, immune cells, vasculature, and the connective tissue stroma, we next wanted to study the expression profiles of complement genes in isolated adipocytes. The levels of 25 out of 45 complement genes expressed by isolated adipocytes differed between heavier and leaner co-twins of the BMI-discordant pairs (Table S3 in Supplementary Material). Similar to AT, in the adipocytes, the complement genes of the classical and alternative pathways were upregulated and genes of the terminal pathway were downregulated in heavier co-twins. However, a few differences in adipocytes compared to the AT expression profile were observed: ADIPOQ, FCN1, CALR, and CFH gene expressions were similar between co-twins of BMI-discordant pairs, but the gene expressions of C1-inhibitor (SERPING1), CD93, C3, CFB, C5AR1, and the MAC-inhibiting molecule CD59 (protectin) were upregulated in heavier co-twins. Again, the gene expression profiles of isolated adipocytes of the co-twins of BMI-concordant pairs were similar (data not shown).

### Adiposity Measures Associate with Upregulated Expression of Genes in the Classical and the Alternative Pathways and Downregulation of Genes in the Terminal Pathway-Related Genes

**Figure 2:**
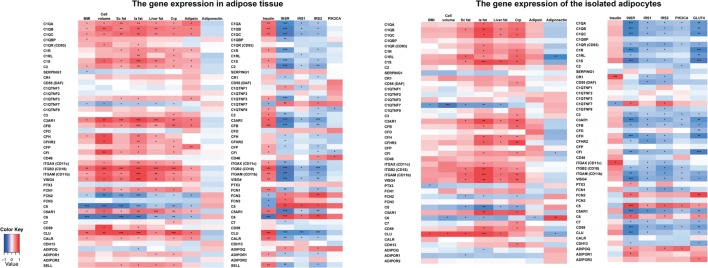
**The correlations between the complement gene expression and the obesity measures, inflammation, and insulin signaling-related genes in monozygotic (MZ) twin individuals taking into account the effect of gender and smoking**. MZ twin individuals, *n* = 80; cell volume, *n* = 65; plasma adipsin, *n* = 70; plasma adiponectin, *n* = 80; insulin, *n* = 80; adipocyte gene transcripts, *n* = 38. *Statistical significance, *p* < 0.05, **p < 0.001, ***p < 0.0001 after multiple correction (false-discovery rate, Benjamini–Hochberg). BMI, Body mass index; Cell volume, adipocyte volume; Sc fat, subcutaneous fat; Ia fat, intra-abdominal fat; Liver fat, liver fat percentage; CRP, high sensitive C-reactive protein; INSR, insulin receptor; IRS1-2, insulin receptor substrate 1-2; PIK3CA, Phosphatidylinositol-4,5-Bisphosphate 3-Kinase, Catalytic Subunit Alpha; GLUT4, glucose transporter type 4.

The correlations between adiposity measures, metabolism, inflammation, and complement gene expression profiles in AT and adipocyte transcripts are presented as heatmaps in Figure 2. Table S4 in Supplementary Material shows the corresponding *r* values and *p* values after correction for multiple parameters.

In AT samples, the complement gene expression levels correlated significantly with BMI (18 out of 46 genes), adipocyte volume (23/46), sc fat (22/46), intra-abdominal fat (29/46), liver fat (22/47), hsCRP (28/46), and plasma adipsin (18/46) levels. The correlations of adiposity measures were positive for genes of the classical and the alternative pathway components and negative for the terminal pathway components C5 and C6. Furthermore, SERPING1 and the C1q homologs C1QTNF7 and FCN2 correlated negatively with adiposity measures.

The complement gene expression of isolated adipocytes correlated most significantly with intra-abdominal fat (24 out of 45 genes), sc and liver fat (13/45 for both), and hsCRP (23/45). The correlations were positive except for C1QTNF7, FCN2, C5, and C6.

### Upregulation of the Classical and Alternative Pathway Components Associates with Hyperinsulinemia and Downregulated Insulin Signaling Route

Next, we examined whether the complement gene expression in AT and adipocytes was associated with plasma insulin and expression of genes along the insulin signaling route (INSR, IRS1–2, and PIK3CA in both AT and adipocytes, and additionally GLUT4 in adipocytes) (Figure 2; Table S4 in Supplementary Material for the corresponding *r* values and *p* values after correction for multiple parameters). A strong inverse correlation between the classical and the alternative pathway and insulin signaling route gene expression was found both in AT and adipocytes. Plasma insulin correlated positively with the expression of 25/46 complement genes in AT but only with CR1 gene expression of adipocytes. C6, C1QTNF7, FCN2, and FCN3 expression in AT correlated negatively with plasma insulin.

### Visualization of Complement Proteins in AT

**Figure 3:**
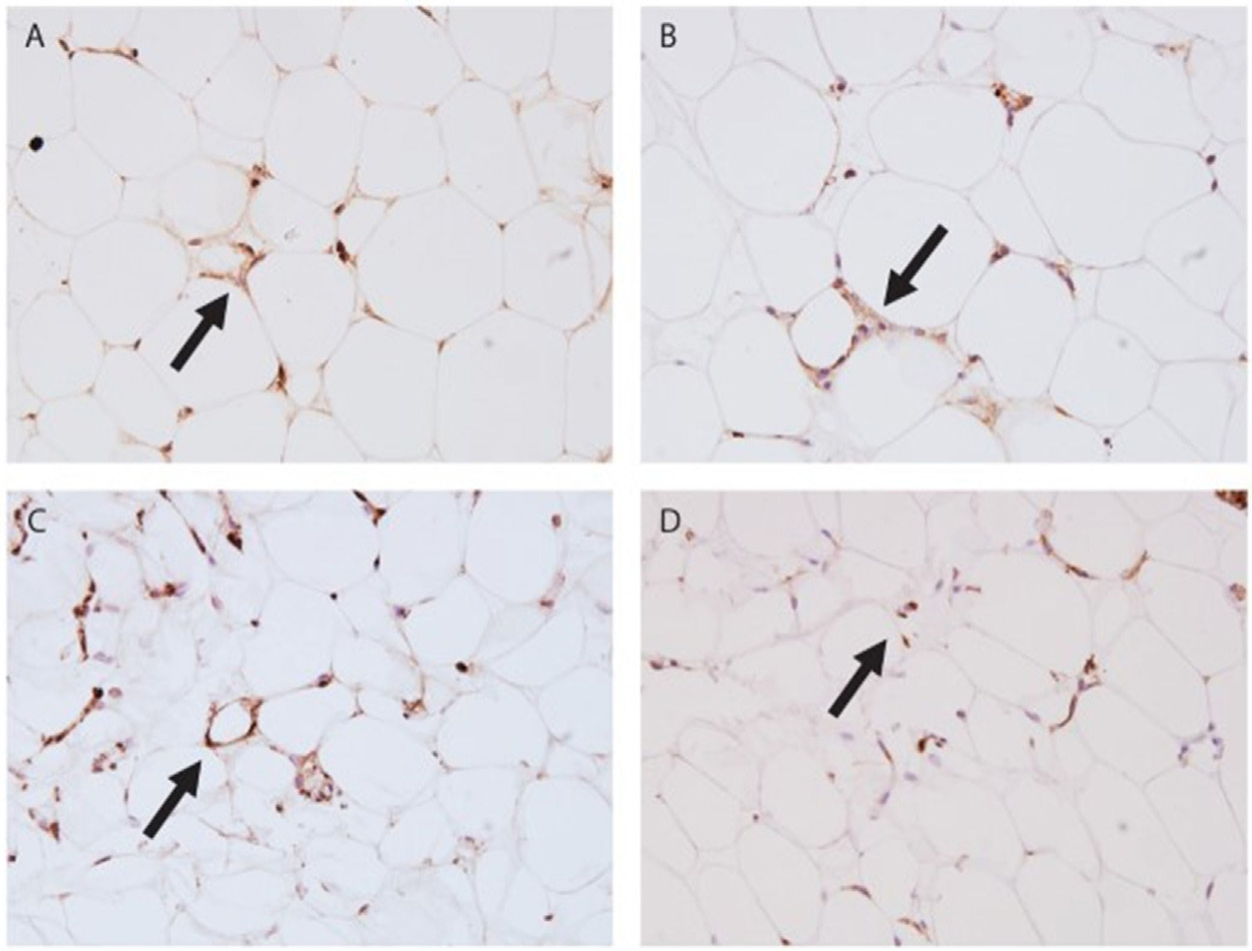
**Immunohistochemistry stain of subcutaneous adipose tissue (AT)**. **(A)** Leaner twin AT stained with C1q (400×); **(B)** leaner twin AT stained with C3d (400×); **(C)** heavier twin AT stained with C1q (400×); **(D)** heavier twin AT stained with C3d (400×); the arrows point out crown-like structures.

Finally, we investigated the location of two complement proteins by immunohistochemistry from paraffin-embedded AT samples. Two complement components were stained: C1q indicating initiation of the classical pathway and C3d representing the remnant of C3b deposition typically observed in prolonged complement activation (examples shown in Figures 3A–D). Crown-like structures representing tissue macrophages around apoptotic adipocytes were observed in both leaner and heavier co-twins of the BMI-discordant pairs, even though they seemed to be more abundant in heavier co-twins’ AT. C1q stained abundantly on cell membranes and intracellularly in the apoptotic cells. Occasionally, intense nuclear staining of adipocytes occurred adjacent but independently of the crown-like structures. C3d stained faintly but evenly on the cell membranes – most likely on the basal surface of the stain-positive cells. Additionally, intracellular granular aggregates staining intensively for C3d emerged in the crown-like structures, but also in some individual adipocytes.

## Discussion

The present study illustrates how obesity can induce a coordinated and synchronized regulation of the complement system genes. The expression of a broad spectrum of complement genes was analyzed in AT and isolated adipocytes in young adult MZ twins, nearly all of whom were free from obesity-related co-morbidities. Within BMI-concordant pairs, the co-twins’ gene-expression profiles were similar suggesting that the overall expression levels of complement genes are largely familial, and probably controlled by genetic or shared environmental factors.

Several interesting differences emerged within BMI-discordant MZ twins: the expression of genes of complement activating and regulating components within AT and isolated adipocytes was clearly different in heavier compared with their leaner co-twins. Most of the differences in the co-twins remained in isolated adipocytes’ transcripts even after separating them from the immune cell-rich stroma-vascular fraction. The main findings were the following: in heavier co-twins, the classical and the alternative pathway complement gene expressions were mostly upregulated. These pathways mediate all main physiological functions of the complement system. They recognize microbes, antigen–antibody complexes, materials from injured cells and tissues, and other targets. Complement activation products promote opsonization, chemotaxis, leukocyte recruitment, activation, and phagocytosis. These effects are mediated by specific receptors that, accordingly, were also upregulated. In line with these results, the plasma levels of C3a, a marker of the activation of early complement pathways, were elevated in the heavier co-twins. Instead, the levels of the soluble terminal pathway complement complex SC5b-9 remained unchanged. This matches with the gene expression data, where the terminal pathway components C5 and C6 were downregulated, while the inhibitor clusterin was upregulated. When combined, the results demonstrate that complement is involved in obesity-related inflammation but not in direct MAC-mediated tissue destruction.

Our results demonstrating the effects of excess body weight in young adults are in accordance with previous studies reporting upregulated complement gene-expression levels in AT in obese, dyslipidemic, and diabetic subjects (4, 21, 23). We made several interesting new observations on upregulated complement genes in the heavier co-twins’ AT and on their association with obesity and metabolic disturbances. All the three sub-chains of C1q, A-C, were upregulated in obesity. They constitute C1q, a multimeric protein that binds to antigen–antibody complexes and to many types of cellular structures, which are either exposed or released during cellular damage (phospholipids, mitochondria, etc.). Bound C1q is subsequently recognized by macrophage receptors that mediate phagocytosis of the targets (34, 35). A novel observation was that the C1r-related protein C1RL gene expression increases in acquired obesity and associates with accumulation of intra-abdominal fat. C1QTNF7, a member of the CTRP family and closely related to C1q, was downregulated in heavier co-twins. Interestingly, C1QTNF7 responds to caloric restriction in mice and therefore has a potential role in the control of the energy balance (36). Furthermore, calreticulin, a C1QR, showed upregulated gene expression in the heavier co-twins and correlated positively with hyperinsulinemia and negatively with the genes involved in the insulin signaling route. The results from Jalali (37) showing the association between INSR density and calreticulin, and Lo (5) demonstrating that calreticulin correlates positively with pancreatic insulin secretion increasing adipsin, support a potential role for calreticulin in the regulation of glucose metabolism.

Another novel observation was that genes of the C3 convertase inhibitor CFH and the alternative pathway suppressing VSIG4 (CRIg), a member of the complement receptor immunoglobulin superfamily, were upregulated in heavier co-twins. VSIG4 correlated strongly positively with overall and intra-abdominal fat and with plasma insulin levels. VSIG4 inhibits T-cell proliferation, clears complement-opsonized particles, and binds to C3b (38). VSIG4 is known as a potential biomarker of severe preeclampsia (39). Therefore, despite the upregulation of complement activating components C3 and CFB in obese subjects, the concomitant upregulation of the alternative pathway regulatory genes indicates that an excessive activation and amplification of this pathway can be suppressed in AT. Alternative pathway activation occurs in a regulated manner generating C3a, and possibly C5a (by the C3/C5 convertases), as well as the C3b inactivation product iC3b (by CFH and CFI), but remains at a reasonable level to prevent transmission of amplification to the terminal pathway.

The levels of SC5b-9, a soluble equivalent of MAC with only one C9 remained unchanged in the heavier co-twins. In the heavier co-twins, upregulation of complement inhibitors and downregulation of the expression of C5 and C6 indicate that excessive early pathway activation in obesity does not translate into the terminal pathway. Thus, complement activation is related more to increased opsonophagocytic and anaphylatoxic capacity than to direct cell damaging activity by MAC. Clusterin gene expression showed overall the most significant differences within BMI-discordant co-twins and the strongest positive correlation between obesity measures, liver fat, hsCRP, hyperinsulinemia, and adipsin and negative correlation with insulin signaling route gene expression in twin individuals. Our results are in line with Won et al. (40), who showed that subjects with metabolic syndrome have elevated levels of clusterin in plasma. In fact, clusterin is a multifunctional molecule, whose activities extend beyond those in the complement system. In addition to being an inhibitor of MAC, it can cluster cells and act as an apolipoprotein, hence the alternative names SP40, 40, and ApoJ (41).

Clusterin suppresses the terminal pathway by inhibiting formation of the complement MAC, composed of components C5b, C6, C7, C8, and multiple C9 molecules (42). Clusterin prevents insertion of intermediate terminal pathway complexes (C5b-7, C5b-8, and C5b-9) to membranes. During this process, it incorporates into the complexes. Increased expression of clusterin may thus be related to the inhibition of excess complement-mediated cell damage that is initiated by increased expression of early pathway components, ischemia, or the release of complement-activating lipids from adipocytes. In addition, by being an apolipoprotein and scavenger for hydrophobic molecules, clusterin could also directly bind to hydrophobic particles released from fat tissue and play a role in their removal.

Adipocyte cell volume was associated with complement gene expression in the AT similarly as the overall obesity measures. A positive association emerged for the adipocyte size with the classical and the alternative pathway and a negative one with the terminal pathway-related genes. The adipocytes undergo changes in their cell membranes in the development of the obese state and potentially this alters the structural conformation and type of surface membrane molecules they express (43). Large adipocytes act as antigen-presenting cells by expressing major histocompatibility class II molecules and take part in the generation of pro-inflammatory immune response (43). Contrasting and potentially generating a balancing response, C1q, a structural homolog of adiponectin, promotes anti-inflammatory macrophage polarization (35, 44, 45). Thus, upregulated C1q expression in AT may be reactive and signal a tissue defense mechanism and local immune cell infiltration. It is noteworthy that multiple C1q homologs, adiponectin, FCN1–2, and C1qTNF7 showed changes in obesity. This suggests their involvement in an adipocyte-macrophage collaboration process, possibly related to debris clearance. As we were limited by sample availability, the immunohistochemical stains did not provide quantifiable data. In the descriptive analyses, a pattern emerged, where C1q stain appeared more intense and widespread in the obese twins than their lean co-twins. The pattern of C1q may reflect ongoing phagocytosis (e.g., of necrotic or apoptotic adipocytes) and/or increased inflammation in the heavier co-twins albeit being present also in the leaner.

The associations between intra-abdominal fat, liver fat, crp, and the genes of the insulin signaling route and differences in most of expression levels of the complement genes remained significant in adipocyte transcripts after the contribution of the stroma-vascular fraction was excluded. In contrast, the role of the stroma may be significant when interpreting the correlations between BMI, sc fat. adipocyte cell size, sc fat and insulin. The correlations between complement gene expressions and these measures were weaker in adipocyte transcripts than in AT. Indeed, it is not the obesity *per se* but the central adiposity and ectopic fat in liver that are related to alterations in the complement gene expression.

Our study of MZ twin pairs is unique in that genetic factors as causes for differences in within-pair comparisons can be excluded. Esparza-Gordillo (24) estimated that 62% of the complement factor H (CFH) phenotypic variation is due to additive genetic effects. Nestvold (46) showed that the complement system is reactive to environmental factors as levels of C3 and C4 dropped after weight loss. The observed differences within weight-discordant pairs emphasize the role of non-genetic influences, including environmental factors and acquired obesity in regulating the expression and activation of the complement system. Finding healthy young adult MZ pairs with significant discordance in body weight is extremely difficult, as body weight is tightly genetically regulated (47). The experiences and exposures of MZ twins are often very similar through childhood and adolescence and begin to differ only after moving out of their parental home and into individual personal and occupational trajectories (48). Therefore, although our sample size is relatively small, the study represents perhaps the best-controlled study design available in humans because of the full match for genes, age, gender, and intrauterine and childhood environment between the lean and heavy groups.

The limitations of our study include that due to small biopsy materials from the volunteers, we lack protein or functional data on the AT samples. However, we documented that circulating C3a protein levels were elevated in obesity, potentially indicating complement activation also at the whole-body level. Our gene-expression analyses also lacked the data for some components of complement cascade such as the pattern recognition protein MBL, C4, C8, and C9. The study setting does not allow direct causal conclusions, but indicates that the observed differences within BMI-discordant co-twins are not due to confounding genetic effects.

In summary, our study shows that characteristic to complement activation in obese AT is upregulation of the classical and the alternative pathway sensors and receptors and downregulation of the terminal pathway. In the AT, the activating factors and the receptors were upregulated simultaneously indicating preparedness for controlled phagocytosis. In addition to the pattern-recognition molecules needed for phagocytic activity, similar gene-expression patterns also emerged in isolated adipocytes, which indicates their independent role in inducing changes in the complement system in the obese AT. Overall, the study reveals the close relationship between the complement system and AT in obesity. These results pave the way for further analyses of the complement-mediated regulation of inflammation and lipid clearance and its potential role leading to adverse metabolic complications in obesity.

## Ethics Statement

The protocol was designed and performed according to the principles of the Helsinki Declaration and was approved by the Ethical Committee of the Helsinki University Central Hospital (DNRO 270/13/03/01/2008).

## Author Contributions

Contributions of the authors were as follows: KP, JK, and AR collected the data. SM, A.Hanttu, and KP designed the studies. SK, KP, and SH performed the clinical studies. AIL performed the immunohistochemistry analyses, EN measured the plasma complement levels and SH calculated the adipocyte volumes. A.Hakkarainen, NL, and JL performed and assisted in measuring the magnetic resonance imaging-related data. OT processed tissue samples for the study. MM and LS assisted in the bioinformatics. SK, A.Hanttu, and SM analyzed and interpreted the data. SK wrote the first draft of the manuscript. SK and AIL edited the final manuscript after all authors had commented and approved the final version.

## Conflict of Interest Statement

The authors declare that the research was conducted in the absence of any commercial or financial relationships that could be construed as a potential conflict of interest.
