# Supplementary material for: Upregulation of Early and Downregulation of Terminal Pathway Complement Genes in Subcutaneous Adipose Tissue and Adipocytes in Acquired Obesity
Source: Front Immunol. 2017 May 16;8:545. doi: 10.3389/fimmu.2017.00545 (PMC5432622; doi:10.3389/fimmu.2017.00545)
Supplement: Supplementary file 1 [file Table_1.DOCX]

**Supplemental Table 1**

| Clinical characteristics of the monozygotic twin pairs, for whom gene expression analyses of isolated adipocytes were available | | | | | | | |
| --- | --- | --- | --- | --- | --- | --- | --- |
|  |  | | | | | |  |
|  | **BMI-discordant pairs,**  **ΔBMI> 3kg/m^2^, n=14 pairs (3 male)** | | | | | **BMI-concordant pairs,**  **ΔBMI < 3kg/m^2^, n=5 pairs (1 male)** | |
| Age 28.90 ± 0.71 | | | | | |  | |
| Smokers 13/38 (34.2%) | | | | | |  | |
|  | **Leaner** | | **Heavier** |  | | **Leaner** | **Heavier** |
|  | **Mean** ± **SE** | | **mean** ± **SE** | **p** | | **mean ±** **SE** | **mean** ± **SE** |
|  |  |  |  |  |  |  |  |
| BMI | 24.07 ± 0.87 | | 29.92 ± 0.98 | 0.001 | | 28.24 ± 1.86 | 30.43 ± 1.77 |
|  |  | |  |  | |  |  |
| Fat percentage (%) | 33.74 ± 1.69 | | 41.95 ± 1.81 | 0.002 | | 35.7 ± 4.85 | 36.88 ± 3.79 |
| Adipocyte volume (um^3^)* | 361.5± 47.2 | | 530.4 ± 67.7 | 0.001 | | 372.6 ± 135.2 | 420.4 ± 105.3 |
| Subcutaneous fat (cm^3^) | 3634 ± 324.7 | | 6020 ± 426.4 | 0.001 | | 4235 ± 668.5 | 4755 ± 1657 |
| Intra-abdominal fat (cm^3^) | 814.3 ±301.3 | | 1504 ±375.8 | 0.002 | | 782.1 ± 293.6 | 954.25 ± 356.4 |
| Liver Fat % | 0.85 ± 0.27 | | 2.96 ±0.81 | 0.003 | | 1.32 ± 0.56 | 2.60 ± 1.82 |
| fP-Insulin mU/L† | 5.01 ± 0.64 | | 8.32 ±1.84 | 0.028 | | 6.06 ± 1.52 | 6.44 ± 0.77 |
| fP-Glucose mmol/L† | 5.07 ± 0.09 | | 5.12 ±0.15 | 0.834 | | 5.30 ± 0.24 | 5.28 ± 0.20 |
| hsCRP mg/L | 3.07 ± 1.10 | | 3.14 ±0.85 | 0.888 | | 0.972 ± 0.35 | 1.37 ± 0.42 |
| Adipsin (pg/L)* | 1181 ± 41.76 | | 1227 ±48.46 | 0.272 | | 1181 ± 314.1 | 1223 ± 133.4 |
| Adiponectin (ng/L) | 2937 ± 343.2 | | 2327 ±269.4 | 0.009 | | 3965 ± 1425 | 2693.1 ± 412.4 |
| fP-C3a (ng/ml) | 74.76 ± 5.54 | | 82.97 ±6.35 | 0.158 | | 72.70 ± 4.21 | 69.62 ± 2.73 |
| fP-SC5b-9 (ng/ml) | 171.2 ± 13.33 | | 182.3 ±11.45 | 0.300 | | 244.96 ± 19.36 | 194.51± 30.57 |
|  |  |  |  |  |  |  |  |

Clinical characteristics of the monozygotic twin pairs in analyses of gene expression of isolated adipocytes. Wilcoxon signed-ranks test (leaner vs heavier twin), smokers included. BMI, body mass index; Δ, within-pair difference; fP, fasting plasma; * n= 3 BMI-concordant pairs; † = 13 BMI-discordant pairs; hsCRP, high-sensitive C-reactive protein, C3a, complement component 3a; SC5b-9, the soluble terminal complement complex (TCC)
