# Supplementary material for: Upregulation of Early and Downregulation of Terminal Pathway Complement Genes in Subcutaneous Adipose Tissue and Adipocytes in Acquired Obesity
Source: Front Immunol. 2017 May 16;8:545. doi: 10.3389/fimmu.2017.00545 (PMC5432622; doi:10.3389/fimmu.2017.00545)
Supplement: Supplementary file 2 [file Table_2.DOCX]

**Supplemental Table 2**

| **The complement gene expression profile in subcutaneous adipose tissue in monozygotic BMI- discordant twin pairs** | | | | | | |  |
| --- | --- | --- | --- | --- | --- | --- | --- |
|  |  |  |  |  |  |  |  |
|  | **Leaner twin** | | **Heavier twin** | |  |  |  |
|  | **Leaner** | | **Heavier** | |  |  |  |
|  | mean | SE | mean | SE | p-value |  |  |
|  |  |  |  |  |  |  |  |
| **Classical pathway** |  |  |  |  |  |  |  |
| C1QA | 7.834 | 0.175 | 8.360 | 0.150 | 0.0001 |  |  |
| C1QB | 8.120 | 0.206 | 8.893 | 0.191 | 0.0001 |  |  |
| C1QC | 8.364 | 0.181 | 9.067 | 0.184 | <0.0001 |  |  |
| C1QBP | 10.522 | 0.054 | 10.595 | 0.045 | 0.1230 |  |  |
| C1QR (CD93) | 11.008 | 0.119 | 11.111 | 0.119 | 0.2733 |  |  |
| C1R | 10.822 | 0.084 | 10.938 | 0.118 | 0.3152 |  |  |
| C1RL | 8.405 | 0.032 | 8.480 | 0.038 | 0.0461 |  |  |
| C1S | 10.406 | 0.065 | 10.682 | 0.081 | 0.0042 |  |  |
| C2 | 3.737 | 0.130 | 4.103 | 0.171 | 0.0171 |  |  |
|  |  |  |  |  |  |  |  |
| **Classical pathway regulators** | |  |  |  |  |  |  |
| SERPING1 | 11.755 | 0.068 | 11.716 | 0.077 | 0.5656 |  |  |
| CR1 | 3.127 | 0.047 | 3.230 | 0.058 | 0.0954 |  |  |
| CD55 (DAF) | 9.270 | 0.138 | 9.288 | 0.179 | 0.9101 |  |  |
| C1QTNF1 | 5.515 | 0.075 | 5.474 | 0.075 | 0.5364 |  |  |
| C1QTNF2 | 4.312 | 0.104 | 4.197 | 0.165 | 0.5000 |  |  |
| C1QTNF3 | 7.105 | 0.231 | 7.241 | 0.281 | 0.6355 |  |  |
| C1QTNF7 | 7.393 | 0.147 | 6.915 | 0.155 | 0.0002 |  |  |
| C1QTNF9 | 5.040 | 0.210 | 5.414 | 0.175 | 0.1010 |  |  |
|  |  |  |  |  |  |  |  |
| **Alternative pathway** |  |  |  |  |  |  |  |
| C3 | 12.723 | 0.076 | 12.819 | 0.113 | 0.3810 |  |  |
| C3AR1 | 7.852 | 0.171 | 8.631 | 0.152 | 0.0001 |  |  |
|  |  |  |  |  |  |  |  |
| **Alternative pathway regulators** | |  |  |  |  |  |  |
| CFB | 6.347 | 0.148 | 6.698 | 0.228 | 0.1378 |  |  |
| CFD | 14.992 | 0.030 | 14.985 | 0.038 | 0.7978 |  |  |
| CFH | 10.988 | 0.086 | 11.249 | 0.130 | 0.0155 |  |  |
| CFHR2 | 5.405 | 0.075 | 5.619 | 0.088 | 0.0014 |  |  |
| CFP | 4.424 | 0.043 | 4.585 | 0.092 | 0.0528 |  |  |
| CFI | 7.305 | 0.093 | 7.495 | 0.127 | 0.0689 |  |  |
| CD46 | 9.501 | 0.064 | 9.501 | 0.064 | 0.8816 |  |  |
| ITGAX (CD11c) | 2.964 | 0.139 | 3.052 | 0.097 | 0.5556 |  |  |
| ITGB2 (CD18) | 5.263 | 0.295 | 6.206 | 0.211 | 0.0019 |  |  |
| ITGAM (CD11b) | 7.698 | 0.181 | 8.320 | 0.177 | 0.0010 |  |  |
| VISG4 (CRIg) | 8.010 | 0.212 | 8.834 | 0.177 | 0.0001 |  |  |
| PTX3 | 3.865 | 0.214 | 3.985 | 0.366 | 0.7650 |  |  |
|  |  |  |  |  |  |  |  |
| **Lectin pathway** |  |  |  |  |  |  |  |
| FCN1 | 2.668 | 0.168 | 3.237 | 0.259 | 0.0418 |  |  |
| FCN2 | 4.360 | 0.223 | 3.253 | 0.178 | 0.0005 |  |  |
| FCN3 | 3.519 | 0.181 | 3.406 | 0.146 | 0.4678 |  |  |
|  |  |  |  |  |  |  |  |
| **Terminal pathway** |  |  |  |  |  |  |  |
| C5 | 6.297 | 0.184 | 5.882 | 0.118 | 0.0024 |  |  |
| C5AR1 | 2.994 | 0.174 | 3.381 | 0.181 | 0.0615 |  |  |
| C6 | 8.635 | 0.298 | 7.609 | 0.253 | 0.0001 |  |  |
| C7 | 10.460 | 0.127 | 10.453 | 0.194 | 0.9660 |  |  |
|  |  |  |  |  |  |  |  |
|  |  |  |  |  |  |  |  |
| **Terminal pathway regulators** | |  |  |  |  |  |  |
| CD59 | 11.107 | 0.039 | 11.151 | 0.040 | 0.1537 |  |  |
| CLU | 11.908 | 0.107 | 12.362 | 0.085 | <0.0001 |  |  |
|  |  |  |  |  |  |  |  |
| **Miscalleneous** |  |  |  |  |  |  |  |
| CALR | 6.549 | 0.066 | 6.677 | 0.072 | 0.0297 |  |  |
| CDH13 | 9.140 | 0.128 | 9.028 | 0.147 | 0.4004 |  |  |
| ADIPOQ | 15.039 | 0.054 | 14.934 | 0.050 | 0.0013 |  |  |
| ADIPOR1 | 9.741 | 0.069 | 9.809 | 0.078 | 0.5146 |  |  |
| ADIPOR2 | 10.814 | 0.066 | 10.813 | 0.065 | 0.9893 |  |  |
| SELL | 3.324 | 0.255 | 3.797 | 0.360 | 0.2263 |  |  |

T test, heavier vs. leaner co-twins (n=26 pairs). The smokers (n=14 individuals) included in the analyses. Values are logarithmic, arbitrary units from Affymetrix chip
