# Supplementary material for: Upregulation of Early and Downregulation of Terminal Pathway Complement Genes in Subcutaneous Adipose Tissue and Adipocytes in Acquired Obesity
Source: Front Immunol. 2017 May 16;8:545. doi: 10.3389/fimmu.2017.00545 (PMC5432622; doi:10.3389/fimmu.2017.00545)
Supplement: Supplementary file 3 [file Table_3.DOCX]

**Supplemental Table 3**

**The complement gene expression profile of isolated adipocytes in monozygotic BMI- discordant twin pairs**

|  | **Leaner twin** | | **Heavier twin** | |  |
| --- | --- | --- | --- | --- | --- |
|  | mean | SE | mean | SE | t-test p-value |
| **Classical pathway** |  |  |  |  |  |
| C1QA | 6.883 | 0.355 | 7.736 | 0.355 | 0.0148 |
| C1QB | 6.268 | 0.368 | 7.505 | 0.380 | 0.0046 |
| C1QC | 7.623 | 0.377 | 8.716 | 0.316 | 0.0059 |
| C1QBP | 10.685 | 0.047 | 10.706 | 0.034 | 0.6802 |
| C1QR (CD93) | 10.127 | 0.271 | 10.498 | 0.253 | 0.0349 |
| C1R | 9.170 | 0.273 | 9.589 | 0.276 | 0.1655 |
| C1RL | 7.087 | 0.061 | 7.340 | 0.068 | 0.0036 |
| C1S | 10.336 | 0.182 | 10.932 | 0.163 | 0.0081 |
| C2 | 2.843 | 0.086 | 2.990 | 0.118 | 0.0637 |
| **Classical pathway regulators** | |  |  |  |  |
| SERPING1 | 10.522 | 0.126 | 10.754 | 0.126 | 0.2564 |
| CR1 | 2.171 | 0.002 | 2.216 | 0.034 | 0.1885 |
| CD55 (DAF) | 9.169 | 0.152 | 9.382 | 0.188 | 0.2041 |
| C1QTNF1 | 7.284 | 0.149 | 7.291 | 0.115 | 0.9536 |
| C1QTNF2 | 2.286 | 0.076 | 2.276 | 0.062 | 0.9304 |
| C1QTNF3 | 4.363 | 0.115 | 4.582 | 0.295 | 0.4270 |
| C1QTNF7 | 7.026 | 0.251 | 6.483 | 0.259 | 0.0107 |
| C1QTNF9 | 3.935 | 0.283 | 4.045 | 0.279 | 0.6617 |
| **Alternative pathway pathway** |  |  |  |  |  |
| C3 | 11.458 | 0.186 | 11.806 | 0.169 | 0.0599 |
| C3AR1 | 5.495 | 0.326 | 6.683 | 0.263 | 0.0019 |
| **Alternative pathway regulators** | |  |  |  |  |
| CFB | 5.485 | 0.200 | 6.014 | 0.279 | 0.0950 |
| CFD | 14.977 | 0.050 | 14.990 | 0.040 | 0.7995 |
| CFH | 8.866 | 0.342 | 9.391 | 0.331 | 0.1555 |
| CFHR2 | 3.741 | 0.165 | 3.980 | 0.102 | 0.0551 |
| CFP | 2.458 | 0.000 | 2.486 | 0.018 | 0.1437 |
| CFI | 6.232 | 0.292 | 6.796 | 0.317 | 0.0804 |
| CD46 | 11.012 | 0.147 | 11.223 | 0.060 | 0.2291 |
| ITGAX (CD11c) | 2.571 | 0.008 | 2.665 | 0.068 | 0.1842 |
| ITGB2 (CD18) | 2.699 | 0.169 | 3.247 | 0.312 | 0.0551 |
| ITGAM (CD11b) | 5.312 | 0.286 | 6.302 | 0.303 | 0.0008 |
| VISG4 (CRIg) | 5.154 | 0.466 | 6.944 | 0.373 | 0.0014 |
| PTX3 | 11.081 | 0.354 | 10.430 | 0.403 | 0.0534 |
| **Lectin pathway** |  |  |  |  |  |
| FCN1 | 2.578 | 0.199 | 2.626 | 0.188 | 0.7710 |
| FCN2 | 5.040 | 0.246 | 4.182 | 0.317 | 0.0132 |
| FCN3 | 3.067 | 0.226 | 2.883 | 0.065 | 0.4202 |
| **Terminal pathway** |  |  |  |  |  |
| C5 | 5.841 | 0.236 | 5.333 | 0.178 | 0.0291 |
| C5AR1 | 5.462 | 0.340 | 6.596 | 0.298 | 0.0005 |
| C6 | 8.466 | 0.268 | 7.736 | 0.288 | 0.1502 |
| C7 | 9.003 | 0.378 | 9.069 | 0.435 | 0.8871 |
| **Terminal pathway regulators** | | |  |  |  |
| CD59 | 10.582 | 0.115 | 10.777 | 0.118 | 0.0686 |
| CLU | 12.671 | 0.112 | 13.112 | 0.104 | 0.0030 |
| **Miscalleneous** |  |  |  |  |  |
| CALR | 7.618 | 0.042 | 7.663 | 0.040 | 0.3711 |
| CDH13 | 7.296 | 0.420 | 7.598 | 0.424 | 0.2227 |
| ADIPOQ | 15.090 | 0.069 | 15.033 | 0.064 | 0.0847 |
| ADIPOR1 | 9.978 | 0.045 | 9.899 | 0.048 | 0.3474 |
| ADIPOR2 | 11.426 | 0.089 | 11.435 | 0.142 | 0.9477 |
|  |  | |  | |  |

**Table Legend**

Ttest (heavier vs. leaner co-twins) n=14 pairs. Smokers (n=13) included in the analyses. Values are logarithmic, arbitrary units from Affymetrix chip.
