# Supplementary material for: Upregulation of Early and Downregulation of Terminal Pathway Complement Genes in Subcutaneous Adipose Tissue and Adipocytes in Acquired Obesity
Source: Front Immunol. 2017 May 16;8:545. doi: 10.3389/fimmu.2017.00545 (PMC5432622; doi:10.3389/fimmu.2017.00545)
Supplement: Supplementary file 4 [file Table_4.PDF]

SUPPLEMENTAL TABLE 4a

The correlation between the complement gene expression profile and adiposity measures, inflammation, metabolism and insulin signaling route-related gene expression in subcutaneous adipose tissue in MZ twin individuals

		BMI		Cell volume		Sc fat		Ia fat		Liver fat		Crp		Adipsin		Adiponectin		Insulin		INSR		IRS1		IRS2		PIK3CA	
		r	p	r	p	r	p	r	p	r	p	r	p	r	p	r	p	r	p	r	p	r	p	r	p	r	p
C1QA		0,299	0,028	0,301	0,0356	0,420	0,0007	0,404	0,001	0,373	0,0027	0,353	0,0068	0,463	0,001	-0,235	0,185	0,439	0,000	-0,546	0,000	-0,407	0,002	-0,331	0,007	-0,010	0,9578
C1QB		0,285	0,032	0,385	0,0056	0,411	0,0015	0,486	0,000	0,426	0,0006	0,402	0,0016	0,404	0,004	-0,183	0,310	0,454	0,000	-0,589	0,000	-0,390	0,003	-0,380	0,002	-0,094	0,5961
C1QC		0,342	0,014	0,321	0,0267	0,464	0,0001	0,489	0,000	0,410	0,0010	0,409	0,0014	0,409	0,004	-0,181	0,310	0,463	0,000	-0,632	0,000	-0,429	0,001	-0,397	0,001	-0,047	0,8048
C1QBP		0,275	0,038	0,219	0,1509	0,232	0,0667	0,229	0,064	0,113	0,4287	0,114	0,4194	-0,139	0,356	0,120	0,547	0,192	0,151	-0,426	0,000	-0,185	0,254	-0,164	0,195	0,045	0,8048
C1QR (CD93)		0,239	0,073	0,300	0,0356	0,151	0,2533	0,247	0,046	0,075	0,6256	0,056	0,7152	-0,114	0,449	-0,017	0,970	0,055	0,696	0,012	0,916	-0,174	0,279	-0,187	0,141	-0,256	0,0773
C1R		0,077	0,601	0,177	0,2360	0,294	0,0227	0,387	0,001	0,349	0,0050	0,307	0,0140	0,293	0,046	-0,002	0,999	0,368	0,002	-0,526	0,000	-0,108	0,431	-0,334	0,007	-0,088	0,5980
C1RL		0,037	0,784	0,188	0,2298	0,224	0,0755	0,392	0,001	0,117	0,4282	0,333	0,0095	0,171	0,265	-0,288	0,127	0,179	0,180	-0,231	0,057	-0,278	0,042	-0,210	0,094	-0,110	0,5745
C1S		0,223	0,096	0,386	0,0056	0,384	0,0019	0,526	0,000	0,424	0,0006	0,313	0,0140	0,306	0,036	-0,245	0,185	0,372	0,002	-0,486	0,000	-0,181	0,258	-0,358	0,004	-0,098	0,5961
C2		0,286	0,032	0,304	0,0354	0,287	0,0261	0,414	0,001	0,343	0,0058	0,317	0,0135	0,266	0,071	-0,173	0,322	0,279	0,023	-0,619	0,000	-0,283	0,042	-0,463	0,000	-0,001	0,9960
SERPING1		-0,311	0,026	-0,231	0,1259	-0,141	0,2774	-0,158	0,207	-0,120	0,4229	-0,017	0,9480	0,202	0,179	-0,175	0,322	-0,136	0,315	0,206	0,092	0,125	0,391	0,165	0,195	0,009	0,9578
CR1		0,053	0,690	0,186	0,2298	0,108	0,4188	0,228	0,064	0,247	0,0566	0,217	0,0922	0,252	0,086	0,053	0,999	0,178	0,180	-0,324		-0,044	0,431	-0,251	0,007	-0,018	0,5980
CD55 (DAF)		-0,111	0,436	-0,011	0,9362	-0,002	0,9858	0,060	0,658	0,100	0,4930	0,013	0,9481	0,161	0,279	0,000	0,999	0,142	0,295	-0,177	0,152	0,113	0,428	-0,089	0,468	0,274	0,0623
C1QTNF1		-0,026	0,841	-0,131	0,3793	0,008	0,9858	-0,064	0,652	-0,115	0,4282	-0,055	0,7152	0,236	0,101	-0,165	0,331	-0,072	0,614	0,240	0,048	-0,312	0,025	0,099	0,427	0,248	0,0884
C1QTNF2		-0,103	0,473	-0,061	0,7274	-0,042	0,7813	-0,018	0,874	0,018	0,9256	-0,017	0,9480	0,113	0,449	0,091	0,648	0,035	0,779	-0,177	0,152	0,087	0,501	-0,080	0,507	0,213	0,1579
C1QTNF3		0,121	0,405	0,132	0,3793	0,263	0,0420	0,294	0,015	0,231	0,0744	0,206	0,1113	0,337	0,020	-0,030	0,912	0,326	0,008	-0,514	0,000	0,018	0,898	-0,322	0,008	0,141	0,4207
C1QTNF7		-0,337	0,015	-0,430	0,0018	-0,333	0,0094	-0,336	0,005	-0,338	0,0063	-0,251	0,0467	-0,086	0,539	0,157	0,344	-0,356	0,004	0,297	0,001	0,191	0,242	0,297	0,015	0,278	0,0623
C1QTNF9		0,115	0,428	0,178	0,2360	0,119	0,3707	0,170	0,174	0,025	0,9042	0,118	0,4065	-0,161	0,279	0,069	0,790	0,327	0,008	-0,083	0,525	0,063	0,616	-0,316	0,009	-0,422	0,0055
C3		0,001	0,992	0,004	0,9362	0,181	0,1622	0,205	0,101	0,178	0,1911	0,336	0,0095	0,260	0,077	-0,062	0,790	0,395	0,001	-0,406	0,001	-0,119	0,406	-0,286	0,020	-0,120	0,5239
C3AR1		0,401	0,003	0,458	0,0010	0,491	0,0000	0,657	0,000	0,511	0,0000	0,454	0,0004	0,428	0,002	-0,188	0,310	0,479	0,000	-0,652	0,000	-0,405	0,002	-0,496	0,000	-0,123	0,5867
CFB		0,198	0,140	0,317	0,0278	0,386	0,0019	0,457	0,000	0,349	0,0050	0,485	0,0001	0,450	0,001	-0,167	0,331	0,487	0,000	-0,551	0,000	-0,138	0,360	-0,419	0,001	-0,148	0,3908
CFD		-0,174	0,202	-0,065	0,7240	-0,096	0,4713	-0,130	0,311	-0,065	0,6779	-0,202	0,1156	0,246	0,090	-0,061	0,790	-0,124	0,363	0,134	0,278	0,145	0,360	0,149	0,228	0,269	0,0623
CFH		0,304	0,026	0,434	0,0018	0,237	0,0646	0,423	0,000	0,315	0,0116	0,145	0,2849	-0,008	0,963	-0,052	0,820	0,159	0,233	-0,282	0,020	-0,101	0,450	-0,282	0,021	-0,295	0,0565
CFHR2		0,208	0,121	0,439	0,0016	0,242	0,0604	0,420	0,000	0,282	0,0259	0,306	0,0140	0,006	0,963	-0,119	0,547	0,161	0,231	-0,241	0,048	-0,139	0,360	-0,160	0,200	-0,307	0,0565
CFP		0,054	0,690	0,140	0,3684	0,201	0,1160	0,345	0,004	0,177	0,1911	0,305	0,0140	0,450	0,001	-0,020	0,970	0,293	0,017	-0,283	0,020	-0,062	0,616	-0,323	0,008	-0,044	0,8048
CFI		0,237	0,074	0,496	0,0003	0,330	0,0096	0,387	0,001	0,233	0,0741	0,320	0,0130	0,101	0,506	0,037	0,884	0,299	0,015	-0,249	0,188	-0,141	0,360	-0,249	0,042	-0,360	0,0274
CD46		0,058	0,688	0,120	0,4221	0,048	0,7586	-0,053	0,677	-0,015	0,9256	-0,062	0,7152	-0,063	0,654	-0,051	0,820	-0,039	0,769	0,146	0,238	0,000	0,997	0,265	0,029	0,337	0,0388
ITGAX (CD11c)		0,241	0,073	0,295	0,0382	0,227	0,0718	0,401	0,001	0,350	0,0050	0,307	0,0140	-0,036	0,810	0,010	0,998	0,324	0,008	-0,445	0,000	-0,098	0,452	-0,377	0,002	-0,203	0,1793
ITGB2 (CD18)		0,468	0,000	0,531	0,0001	0,529	0,0000	0,667	0,000	0,544	0,0000	0,535	0,0000	0,338	0,020	-0,183	0,310	0,518	0,000	-0,662	0,000	-0,392	0,003	-0,525	0,000	-0,213	0,1579
ITGAM (CD11b)		0,305	0,026	0,397	0,0045	0,394	0,0015	0,576	0,000	0,469	0,0002	0,373	0,0038	0,250	0,086	-0,090	0,648	0,425	0,000	-0,641	0,000	-0,283	0,042	-0,465	0,000	-0,134	0,4427
VISG4		0,366	0,008	0,428	0,0018	0,447	0,0002	0,605	0,000	0,473	0,0002	0,400	0,0016	0,326	0,023	-0,115	0,547	0,412	0,001	-0,608	0,000	-0,350	0,010	-0,408	0,001	-0,082	0,6223
PTX3		-0,141	0,324	-0,183	0,2325	-0,145	0,2719	-0,118	0,358	0,007	0,9490	0,008	0,9481	0,095	0,527	-0,182	0,310	-0,023	0,841	0,050	0,697	0,073	0,575	0,119	0,337	0,152	0,3908
FCN1		0,191	0,156	0,323	0,0266	0,263	0,0420	0,376	0,001	0,380	0,0023	0,421	0,0010	0,164	0,279	-0,102	0,624	0,414	0,001	-0,409	0,000	-0,150	0,360	-0,396	0,001	-0,167	0,3908
FCN2		-0,290	0,032	-0,518	0,0002	-0,498	0,0000	-0,531	0,000	-0,310	0,0126	-0,261	0,0405	-0,447	0,001	0,294	0,127	-0,312	0,011	0,285	0,020	0,257	0,067	0,422	0,001	0,268	0,0623
FCN3		-0,076	0,601	-0,037	0,8266	-0,254	0,0498	-0,175	0,164	-0,216	0,0984	-0,241	0,0576	-0,273	0,069	0,236	0,185	-0,394	0,001	0,312	0,010	0,150	0,360	0,150	0,228	-0,095	0,5961
C5		-0,625	0,000	-0,611	0,0000	-0,608	0,0000	-0,487	0,000	-0,321	0,0102	-0,459	0,0004	-0,336	0,020	0,297	0,127	-0,397	0,001	0,409	0,000	0,440	0,001	0,371	0,002	0,318	0,0518
C5AR1		0,283	0,032	0,382	0,0057	0,268	0,0410	0,459	0,000	0,447	0,0004	0,335	0,0095	0,269	0,070	-0,235	0,185	0,439	0,000	-0,596	0,000	-0,281	0,042	-0,453	0,000	-0,026	0,8963
C6		-0,617	0,000	-0,570	0,0000	-0,668	0,0000	-0,597	0,000	-0,426	0,0006	-0,425	0,0010	-0,387	0,006	0,252	0,185	-0,539	0,000	0,458	0,000	0,312	0,025	0,436	0,000	0,299	0,0565
C7		-0,065	0,655	-0,056	0,7451	-0,002	0,9858	0,066	0,648	0,038	0,8545	0,253	0,0467	0,086	0,539	-0,094	0,648	0,084	0,553	-0,277	0,022	-0,131	0,391	-0,052	0,664	-0,055	0,8048
CD59		0,141	0,324	0,445	0,0015	0,151	0,2533	0,307	0,011	0,134	0,3585	0,010	0,9481	-0,088	0,539	0,047	0,827	0,093	0,511	-0,055	0,696	-0,164	0,314	-0,166	0,195	-0,107	0,5745
CLU		0,484	0,000	0,468	0,0009	0,688	0,0000	0,641	0,000	0,395	0,0016	0,568	0,0000	0,460	0,001	-0,346	0,093	0,665	0,000	-0,538	0,000	-0,538	0,000	-0,515	0,000	-0,268	0,0623
CALR		0,309	0,026	0,198	0,2031	0,314	0,0142	0,246	0,046	0,160	0,2481	0,307	0,0140	0,364	0,011	-0,257	0,185	0,457	0,000	-0,451	0,000	-0,283	0,042	-0,324	0,008	-0,051	0,8048
CDH13		0,083	0,584	0,134	0,3793	0,014	0,9635	0,058	0,658	-0,079	0,6089	0,109	0,4319	-0,173	0,265	0,227	0,196	0,101	0,476	0,050	0,697	0,124	0,391	0,011	0,927	-0,229	0,1261
ADIPOQ		-0,251	0,065	-0,171	0,2506	-0,234	0,0667	-0,312	0,010	-0,013	0,9256	-0,187	0,1465	-0,145	0,339	0,158	0,344	-0,041	0,769	0,249	0,042	0,127	0,391	0,278	0,022	0,291	0,0565
ADIPOR1		0,245	0,071	0,046	0,7865	0,247	0,0564	0,190	0,129	0,274	0,0304	0,269	0,0350	0,174	0,265	-0,184	0,310	0,342	0,005	-0,341	0,004	-0,197	0,226	-0,281	0,021	-0,037	0,8400
ADIPOR2		0,139	0,324	0,023	0,8969	0,095	0,4713	0,020	0,874	0,026	0,9042	0,067	0,7152	-0,236	0,098	0,062	0,790	0,055	0,696	0,026	0,839	-0,101	0,449	0,132	0,282	-0,013	0,9578
SELL		0,221	0,097	0,248	0,0960	0,313	0,0142	0,382	0,0012	0,384	0,0022	0,348	0,0073	0,116	0,4493	-0,115	0,547	0,440	0,000	-0,449	0,000	-0,145	0,360	-0,411	0,001	-0,150	0,3908

Table Legend

The partial correlations between the complement gene expression and measures of obesity, inflammation, metabolism and the expression of insulin signalling related genes in monozygotic twin individuals taking account for the effect of smoking and gender.

Monozygotic twin individuals n=81; Cell volume n=66, Plasma adipsin n=70; Plasma Adiponectin n=80, P-values after multiple correction (FDR, Benjamini-Hochberg)

BMI, Body mass index; Cell volume, adipocyte volume; Sc fat, subcutaneous fat; Ia fat , intra-abdominal fat; Liver fat, liver fat percentage; CRP, high sensitive C-reactive protein; INSR, insulin receptor; IRS1-2, insulin receptor substrate 1-2; PIK3CA, Phosphatidylinositol-4,5-Bisphosphate 3-Kinase, Catalytic Subunit Alpha; GLUT4, glucose transporter type 4

SUPPLEMENTAL TABLE 4b

The correlations between the complement gene expression profile and adiposity measures, inflammation, metabolism and insulin signaling route-related gene expression of isolated adipocytes in MZ twin individuals

		BMI		Cell volume		Sc fat		Ia fat		Liver fat		Crp		Adipsin		Adiponectin		Insulin		INSR		IRS1		IRS2		PIK3CA		GLUT4	
		r	p	r	p	r	p	r	p	r	p	r	p	r	p	r	p	r	p	r	p	r	p	r	p	r	p	r	p
C1QA		0,151	0,669	0,129	0,6813	0,301	0,1759	0,502	0,0054	0,382	0,064	0,457	0,0209	0,215	0,6091	-0,325	0,258	0,190	0,3985	-0,673	0,0000	-0,497	0,0071	-0,429	0,0185	-0,4	0,0473	-0,617	0,000

C1QB	0,278	0,323	0,257	0,3332	0,42	0,0372	0,601	0,0005	0,427	0,0423	0,485	0,0128	0,202	0,6523	-0,363	0,201	0,258	0,3249	-0,687	0,0000	-0,493	0,0072	-0,509	0,0057	-0,433	0,04	-0,61	0,000
C1QC	0,227	0,458	0,215	0,4480	0,395	0,0555	0,575	0,001	0,422	0,0423	0,523	0,0081	0,239	0,5383	-0,364	0,201	0,278	0,2976	-0,672	0,0000	-0,519	0,0071	-0,433	0,0178	-0,374	0,0652	-0,631	0,000
C1QBP	0,131	0,720	0,040	0,9757	0,135	0,6081	0,297	0,1075	0,133	0,5635	0,176	0,401	-0,087	0,727	-0,097	0,730	0,157	0,4979	-0,296	0,1073	-0,148	0,4603	-0,181	0,3366	-0,207	0,2979	-0,018	0,920
C1QR (CD93)	-0,008	0,977	0,146	0,6679	0,252	0,2694	0,428	0,0228	0,324	0,1059	0,352	0,0664	0,155	0,684	-0,392	0,201	0,203	0,3985	-0,497	0,0044	-0,521	0,0071	-0,543	0,0035	-0,402	0,0473	-0,525	0,003
C1R	0,060	0,860	-0,018	0,9788	0,165	0,4877	0,445	0,0173	0,364	0,0726	0,455	0,0209	0,256	0,5203	-0,242	0,387	0,231	0,3276	-0,666	0,0000	-0,437	0,0189	-0,391	0,0317	-0,326	0,1116	-0,691	0,000
C1RL	0,267	0,332	0,301	0,2710	0,516	0,0143	0,390	0,037	0,251	0,2165	0,496	0,0115	0,382	0,3483	-0,644	0,010	0,234	0,3276	-0,471	0,0076	-0,306	0,1162	-0,163	0,3655	-0,123	0,5626	-0,607	0,000
C1S	0,321	0,212	0,346	0,1886	0,425	0,0372	0,690	0,0001	0,576	0,0095	0,537	0,0081	0,260	0,5203	-0,517	0,055	0,434	0,0835	-0,781	0,0000	-0,506	0,0071	-0,479	0,0093	-0,3	0,1333	-0,746	0,000
C2	0,115	0,723	-0,009	0,9789	0,165	0,4877	0,351	0,0617	0,097	0,6814	0,171	0,401	-0,117	0,684	0,211	0,404	-0,007	0,9677	-0,341	0,0612	-0,23	0,2648	-0,301	0,1086	-0,448	0,0398	-0,044	0,899
SERPING1	-0,441	0,064	-0,468	0,0536	-0,246	0,2777	-0,361	0,0554	-0,334	0,0955	-0,028	0,9168	-0,038	0,8959	-0,122	0,672	-0,245	0,3276	0,156	0,4191	-0,102	0,6073	0,238	0,1969	-0,32	0,1116	-0,22	0,296
CR1	0,149	0,669	0,378	0,1497	0,313	0,1585	0,344	0,0669	0,266	0,1957	0,33	0,0819	-0,109	0,684	0,112	0,661	0,605	0,0027	-0,077	0,6839	-0,443	0,0189	-0,488	0,0081	-0,251	0,2102	-0,036	0,917
CD55 (DAF)	-0,020	0,952	-0,020	0,9788	0,084	0,7246	0,333	0,0733	0,221	0,2936	0,38	0,0475	0,020	0,9336	-0,079	0,776	0,237	0,3276	-0,256	0,1698	-0,557	0,0054	-0,510	0,0057	-0,361	0,0768	-0,344	0,070
C1QTNF1	0,078	0,813	-0,223	0,4324	0,047	0,8441	0,149	0,4141	-0,033	0,8579	0,184	0,3836	-0,159	0,684	-0,021	0,916	0,182	0,4163	-0,169	0,3827	-0,151	0,4603	0,228	0,2135	-0,027	0,3485	-0,138	0,544
C1QTNF2	-0,151	0,669	-0,159	0,6598	-0,084	0,7246	-0,044	0,8374	0,115	0,6284	0,122	0,5603	0,151	0,684	0,224	0,391	-0,019	0,9571	-0,069	0,7072	0,155	0,458	0,024	0,8899	0,067	0,7677	-0,084	0,725
C1QTNF3	0,217	0,482	-0,026	0,9788	0,165	0,4877	0,176	0,3524	0,047	0,8408	-0,17	0,401	0,326	0,3764	0,209	0,404	0,085	0,7708	-0,144	0,4465	0,03	0,9043	-0,257	0,1678	0,039	0,859	-0,14	0,544
C1QTNF7	-0,597	0,004	-0,766	0,0000	-0,582	0,0044	-0,591	0,0007	-0,548	0,0095	-0,299	0,1217	-0,101	0,684	0,272	0,317	-0,560	0,0071	0,550	0,0014	0,162	0,4523	0,549	0,0034	0,237	0,2315	0,15	0,521
C1QTNF9	-0,103	0,752	-0,060	0,9536	0,113	0,6408	0,222	0,2426	-0,031	0,8579	0,355	0,0656	-0,037	0,8959	0,231	0,391	0,154	0,4979	-0,226	0,2245	-0,313	0,1099	-0,460	0,0112	-0,322	0,1116	-0,211	0,306
C3	0,032	0,912	-0,136	0,6813	0,052	0,8382	0,321	0,0822	0,281	0,1751	0,391	0,0439	-0,105	0,684	0,130	0,672	0,194	0,3985	-0,519	0,0028	-0,35	0,0473	-0,373	0,0421	-0,452	0,0398	-0,374	0,046
C3AR1	0,288	0,307	0,281	0,2823	0,441	0,0323	0,611	0,0004	0,424	0,0423	0,524	0,0081	0,269	0,5203	-0,317	0,266	0,256	0,3249	-0,681	0,0000	-0,511	0,0071	-0,536	0,0037	-0,415	0,041	-0,633	0,000
CFB	0,112	0,723	0,127	0,6813	0,354	0,0966	0,425	0,023	0,256	0,2112	0,494	0,0115	0,357	0,3483	-0,309	0,273	0,299	0,2433	-0,552	0,0014	-0,499	0,0071	-0,413	0,0232	-0,456	0,0398	-0,585	0,001
CFD	-0,005	0,977	-0,035	0,9788	0,117	0,6408	0,032	0,8523	0,092	0,6833	0,013	0,9648	0,462	0,1668	-0,495	0,067	-0,049	0,8771	-0,295	0,1073	0,051	0,8231	0,136	0,4373	-0,013	0,9413	-0,462	0,010
CFH	0,092	0,761	0,108	0,7484	0,178	0,4786	0,505	0,0054	0,412	0,0461	0,413	0,0366	0,195	0,6565	-0,470	0,088	0,257	0,3249	-0,640	0,0001	-0,552	0,0054	-0,450	0,0133	-0,328	0,1116	-0,69	0,000
CFHR2	0,152	0,669	0,300	0,2710	0,23	0,3083	0,532	0,0031	0,409	0,0461	0,599	0,0065	-0,108	0,684	-0,325	0,258	0,378	0,1428	-0,378	0,0368	-0,617	0,0014	-0,501	0,0064	-0,164	0,4114	-0,328	0,084
CFP	0,144	0,670	-0,045	0,9757	0,114	0,6408	0,188	0,3219	0,099	0,6814	0,006	0,9749	0,357	0,3483	0,042	0,892	0,031	0,9264	-0,143	0,4465	0,008	0,9617	-0,154	0,387	0,021	0,9259	-0,086	0,725
CFI	0,047	0,904	0,167	0,6589	0,256	0,2694	0,392	0,037	0,367	0,0726	0,409	0,0366	0,311	0,3897	-0,435	0,134	0,192	0,3985	-0,605	0,0003	-0,498	0,0071	-0,469	0,0097	-0,306	0,1308	-0,729	0,000
CD46	0,360	0,166	0,283	0,2823	0,176	0,284	0,287	0,1181	0,277	0,1765	0,093	0,6698	0,155	0,684	-0,300	0,273	0,360	0,152	0,005	0,9771	-0,302	0,1173	-0,175	0,3385	0,284	0,1551	-0,017	0,920
ITGAX (CD11c)	0,205	0,518	0,422	0,0935	0,339	0,1148	0,396	0,037	0,35	0,0844	0,337	0,0764	-0,109	0,684	-0,028	0,910	0,636	0,0018	-0,126	0,4985	-0,404	0,03	-0,476	0,0095	-0,212	0,2913	-0,026	0,920
ITGB2 (CD18)	0,344	0,180	0,423	0,0935	0,449	0,0299	0,653	0,0001	0,434	0,0423	0,38	0,0475	0,021	0,9336	-0,052	0,869	0,442	0,0835	-0,467	0,0081	-0,435	0,0189	-0,610	0,0021	-0,432	0,04	-0,217	0,296
ITGAM (CD11b)	0,330	0,203	0,344	0,1886	0,497	0,0153	0,681	0,0001	0,457	0,0381	0,524	0,0081	0,105	0,684	-0,226	0,391	0,360	0,152	-0,624	0,0002	-0,486	0,0081	-0,584	0,0021	-0,416	0,041	-0,537	0,002
VISG4	0,356	0,166	0,271	0,3015	0,474	0,0195	0,640	0,0002	0,432	0,0423	0,516	0,0085	0,146	0,684	-0,265	0,326	0,313	0,2155	-0,686	0,0000	-0,514	0,0071	-0,533	0,0037	-0,43	0,04	-0,583	0,001
PTX3	-0,489	0,037	-0,352	0,1886	-0,288	0,1902	-0,313	0,0893	-0,217	0,2946	0,293	0,1279	-0,284	0,4896	0,115	0,683	-0,014	0,9596	0,388	0,0337	-0,105	0,6073	0,350	0,0587	-0,061	0,7757	0,028	0,920
FCN1	-0,043	0,904	0,331	0,2086	0,099	0,6887	0,328	0,076	0,301	0,1393	0,391	0,0439	-0,170	0,684	-0,291	0,277	0,323	0,2025	-0,346	0,0579	-0,433	0,0189	-0,591	0,0021	-0,455	0,0398	-0,236	0,259
FCN2	-0,198	0,528	-0,248	0,3534	-0,491	0,0153	-0,389	0,037	-0,348	0,0844	-0,39	0,0439	-0,349	0,3483	0,363	0,201	-0,245	0,3276	0,535	0,0020	0,203	0,3194	0,340	0,0661	0,322	0,1116	0,644	0,000
FCN3	-0,032	0,912	-0,015	0,9788	0,028	0,9112	0,167	0,3726	0,055	0,822	0,235	0,2454	0,185	0,68	-0,032	0,910	-0,130	0,9596	-0,229	0,0337	-0,123	0,6073	-0,263	0,0587	-0,199	0,7757	-0,099	0,920
C5	-0,402	0,098	-0,478	0,0536	-0,544	0,0091	-0,669	0,0001	-0,475	0,0306	-0,538	0,0081	-0,125	0,684	0,371	0,201	-0,378	0,1428	0,763	0,0000	0,396	0,0327	0,560	0,0031	0,504	0,0387	0,393	0,035
C5AR1	0,265	0,332	0,292	0,2778	0,371	0,0783	0,627	0,0003	0,523	0,0855	0,562	0,0081	0,103	0,684	-0,368	0,201	0,349	0,1587	-0,743	0,0000	-0,634	0,0014	-0,589	0,0021	-0,417	0,041	-0,569	0,001
C6	-0,465	0,048	-0,565	0,0092	-0,507	0,0143	-0,511	0,005	-0,532	0,0095	-0,203	0,3295	-0,458	0,1668	0,621	0,010	-0,345	0,1587	0,553	0,0014	0,218	0,2916	0,392	0,0317	0,067	0,7677	0,416	0,024
C7	-0,095	0,761	-0,153	0,6598	-0,065	0,7955	0,232	0,2222	0,207	0,3068	0,409	0,0366	0,001	0,9957	-0,095	0,730	0,083	0,7708	-0,458	0,0093	-0,43	0,0189	-0,280	0,1375	-0,389	0,0539	-0,493	0,006
CD59	0,115	0,723	0,152	0,6598	0,291	0,1902	0,494	0,0062	0,375	0,0687	0,421	0,0355	0,243	0,5383	-0,333	0,258	0,235	0,3276	-0,692	0,0000	-0,499	0,0071	-0,558	0,0031	-0,448	0,0398	-0,581	0,001
CLU	0,584	0,004	0,614	0,0032	0,641	0,0012	0,707	0,0001	0,538	0,0095	0,338	0,0764	0,143	0,684	-0,528	0,055	0,414	0,101	-0,690	0,0000	-0,432	0,0189	-0,413	0,0232	-0,26	0,1951	-0,44	0,016
CALR	0,258	0,342	-0,004	0,9826	0,002	0,9914	0,155	0,4013	0,166	0,4413	-0,087	0,6806	-0,127	0,684	0,125	0,672	0,030	0,9264	-0,361	0,0472	0,018	0,9367	-0,239	0,1969	-0,273	0,1724	0,087	0,725
CDH13	-0,155	0,669	-0,079	0,8742	0,119	0,6408	0,216	0,2514	0,214	0,2946	0,448	0,0223	0,233	0,5383	-0,289	0,277	0,195	0,3985	-0,379	0,0368	-0,354	0,062	-0,313	0,0947	-0,299	0,1333	-0,554	0,001
ADIPOQ	-0,065	0,858	-0,126	0,6813	-0,184	0,4733	-0,379	0,0427	-0,072	0,7632	-0,146	0,4755	-0,041	0,8959	0,304	0,273	-0,079	0,7746	0,400	0,0281	0,291	0,133	0,472	0,0096	0,537	0,0333	0,24	0,254
ADIPOR1	-0,419	0,082	-0,384	0,1497	-0,423	0,0372	-0,341	0,0673	-0,387	0,0639	-0,373	0,0511	-0,323	0,3764	0,217	0,401	-0,197	0,3985	0,503	0,004	0,209	0,3116	0,175	0,3385	0,081	0,7378	0,357	0,059
ADIPOR2	0,120	0,723	-0,048	0,9757	-0,016	0,9455	-0,036	0,8498	-0,037	0,8579	-0,027	0,9168	-0,124	0,684	0,220	0,391	0,073	0,7783	0,295	0,1073	0,158	0,4523	0,263	0,1582	0,239	0,2315	0,471	0,009

Table Legend

The correlations between the complement gene expression and measures of obesity, inflammation, metabolism and the expression of insulin signalling related genes in monozygotic twin individuals taking account for the effect of smoking and gender.

Monozygotic twin individuals n=38; Insulin n=37, P-values after multiple correction (FDR, Benjamini-Hochberg)

BMI, Body mass index; Cell volume, adipocyte volume; Sc fat, subcutaneous fat; Ia fat , intra-abdominal fat; Liver fat, liver fat percentage; CRP, high sensitive C-reactive protein; INSR, insulin receptor; IRS1-2, insulin receptor substrate 1-2; PIK3CA, Phosphatidylinositol-4,5-Bisphosphate 3-Kinase, Phosphatidylinositol-4,5-Bisphosphate 3-K Catalytic Subunit Alpha; GLUT4, glucose transporter type 4
